# 572 Trace Element Supplementation in Burn Patients: A Quality Improvement Project

**DOI:** 10.1093/jbcr/irad045.167

**Published:** 2023-05-15

**Authors:** Alexander Kurjatko, Isaac Weigel, Colette Galet, Justin Suarez, Kelly Cavanaugh, Lucy Wibbenmeyer

**Affiliations:** University of Iowa, Iowa City, Iowa; University of Iowa, Iowa City, Iowa; University of Iowa, Iowa City, Iowa; University of Iowa, Iowa City, Iowa; University of Iowa, Iowa City, Iowa; University of Iowa, Iowa City, Iowa; University of Iowa, Iowa City, Iowa; University of Iowa, Iowa City, Iowa; University of Iowa, Iowa City, Iowa; University of Iowa, Iowa City, Iowa; University of Iowa, Iowa City, Iowa; University of Iowa, Iowa City, Iowa; University of Iowa, Iowa City, Iowa; University of Iowa, Iowa City, Iowa; University of Iowa, Iowa City, Iowa; University of Iowa, Iowa City, Iowa; University of Iowa, Iowa City, Iowa; University of Iowa, Iowa City, Iowa; University of Iowa, Iowa City, Iowa; University of Iowa, Iowa City, Iowa; University of Iowa, Iowa City, Iowa; University of Iowa, Iowa City, Iowa; University of Iowa, Iowa City, Iowa; University of Iowa, Iowa City, Iowa; University of Iowa, Iowa City, Iowa; University of Iowa, Iowa City, Iowa; University of Iowa, Iowa City, Iowa; University of Iowa, Iowa City, Iowa; University of Iowa, Iowa City, Iowa; University of Iowa, Iowa City, Iowa; University of Iowa, Iowa City, Iowa; University of Iowa, Iowa City, Iowa; University of Iowa, Iowa City, Iowa; University of Iowa, Iowa City, Iowa; University of Iowa, Iowa City, Iowa; University of Iowa, Iowa City, Iowa

## Abstract

**Introduction:**

Patients with major burns suffer deficiencies in trace elements (TE), which can affect their clinical course. We established a quality improvement (QI) project to supplement TE (multivitamins with TE, vitamin C, and zinc). Herein, we assessed the impact of QI implementation on outcomes.

**Methods:**

We queried the burn registry for burn patients admitted from 8/1/2019-5/31/2022 who weighed > 40 Kg and stayed >14 days. Demographics; comorbidities; injury, and hospital course information were collected. Post-implementation, TE levels (zinc, copper, and selenium) as well as C reactive protein levels were measured at 2 weeks. Analyses were performed to assess differences between the pre- and post-groups and post-group patients with major burns (≥20%TBSA) and smaller burns (< 20%TBSA). P < 0.05 was considered significant.

**Results:**

We included 111 patients, 45 in the pre- and 66 in the post-group. Overall, the population was male (79.3%) with a median age of 53 years. Age and sex were not significantly different between the pre- and post-groups. The post-group had higher TBSA (p = 0.005). They were more likely to smoke (p =0.018) and have an alcohol use disorder (p = 0.035). There was no significant difference observed in mortality, complications, or LOS/TBSA (2.1 days [1.2-5.5] vs. 1.7 days [1.2-3.1], p = 0.300) between the groups. Focusing on the post-group, 37 presented with smaller burns and 29 with major burns. Patients with major burns were younger (p =0.003) and healthier than patients with smaller burns. At 14 days, 81.5% and 33.3% of patients with major burns were deficient in zinc and copper, respectively. Zinc (47.6 µg/dL vs.79.2 µg/dL p < 0.001), copper (80.2 µg/dL vs.121.9 µg/dL p < 0.001), and selenium (98.7 µg/L vs.135.5 µg/L, p = 0.003) levels were significantly lower in patients with major burns. C-reactive protein levels were high in both groups but significantly higher in patients with major burns (p < 0.001). Patients with major burns were more likely to have infectious complications (p = 0.004); however their LOS/TBSA was significantly lower than that of patients with smaller burns (1.21 days vs. 2.43 days, p < 0.001). Zinc levels of patients with major burns significantly increased overtime from 47.6 µg/dL at 14 days post-admission to 73.5 µg/dL at 53 ± 16 days (p < 0.01).

**Conclusions:**

Our data show that TE supplementation was efficient in bringing zinc levels close to the normal range in patients with burns ≥20%TBSA who stayed up to 50 days in hospital. Controlled for TBSA burned, supplementation of TE significantly decreased LOS of patients with major burns compared to patients with smaller burns.

**Applicability of Research to Practice:**

Further studies are needed to evaluate the correlation between supplementation of TE and improvement of patient LOS.

